# Implementing Oregon’s New Quality Measurement Program for Assisted Living Communities

**DOI:** 10.1093/geroni/igaa057.2577

**Published:** 2020-12-16

**Authors:** Paula Carder, Ann McQueen, Ozcan Tunalilar

**Affiliations:** 1 Portland State University, Portland, Oregon, United States; 2 Oregon Department Human Services, Salem, Oregon, United States

## Abstract

Oregon legislation requires assisted living (AL) communities to report selected quality measures to the state licensing agency. The Quality Measurement Program (QMP) includes five metrics that assess different areas of resident safety and wellbeing: falls, antipsychotic medication use, staff training, staff retention, and resident satisfaction. This paper describes findings based on our 2019 survey of AL communities and offers suggestions for stakeholders interested in public reporting and quality metrics. Assisted living providers reported 28% of AL residents and 38% of memory care (MC) residents fell at least once in the prior 90 days, with 39% and 45% reporting an injury, respectively. Antipsychotic medication use was 20% among AL and 44% among MC residents. These findings and the survey methods used to collect them, combined with stakeholder and state agency staff participation, informed the current QMP approach. We describe how to collect meaningful quality metrics within the AL context.

